# The Efficacy of Plant-Based Bioactives Supplementation to Different Proportion of Concentrate Diets on Methane Production and Rumen Fermentation Characteristics In Vitro

**DOI:** 10.3390/ani11041029

**Published:** 2021-04-05

**Authors:** Eslam Ahmed, Naoki Fukuma, Masaaki Hanada, Takehiro Nishida

**Affiliations:** 1 Graduate School of Animal Husbandry, Obihiro University of Agriculture and Veterinary Medicine, Inada, Obihiro 080-8555, Japan; eslam_kh@vet.svu.edu.eg; 2 Department of Animal Behavior and Management, Faculty of Veterinary Medicine, South Valley University, Qena 83523, Egypt; 3 Department of Life and Food Sciences, Obihiro University of Agriculture and Veterinary Medicine, Inada, Obihiro 080-8555, Japan; n.fukumax@obihiro.ac.jp (N.F.); hanada@obihiro.ac.jp (M.H.); 4 Research Center for Global Agromedicine, Obihiro University of Agriculture and Veterinary Medicine, Inada, Obihiro 080-8555, Japan

**Keywords:** garlic, citrus, methane emission, rumen fermentation, digestibility

## Abstract

**Simple Summary:**

Using natural feed additives to mitigate methane emissions from ruminants is a promising strategy. Many antimethanogenic compounds have been used to alter rumen fermentation, yet their potential to reduce methane production effectively is not consistent across different kinds of feeding styles (forage:concentrate ratios). Consequently, in the current study we investigated the efficacy of plant-bioactives extract (PE) (a novel phytogenic mixture of garlic and citrus extracts) on rumen fermentation characteristics and methane production in different kinds of feeding styles. The current In Vitro study showed that PE was effective in reducing methane production in all feeding styles without exhibiting any adverse effect on nutrient digestibility. Furthermore, PE supplementation was able to improve the rumen fermentation through increasing the production of total volatile fatty acids. Therefore, PE mixture could be used as a dietary supplement to reduce the methane production from ruminants.

**Abstract:**

This In Vitro study was conducted to investigate the impact of plant-bioactives extract (PE), a combination of garlic powder and bitter orange extract, on methane production, rumen fermentation, and digestibility in different feeding models. The dietary treatments were 1000 g grass/kg ration + 0 g concentrate/kg ration (100:0), 80:20, 60:40, 40:60, and 20:80. The PE was supplemented at 200 g/kg of the feed. Each group consisted of 6 replicates. The experiment was performed as an In Vitro batch culture for 24 h at 39 °C. This procedure was repeated in three consecutive runs. The results of this experiment showed that supplementation with PE strongly reduced methane production in all kinds of feeding models (*p* < 0.001). Its efficacy in reducing methane/digestible dry matter was 44% in the 100:0 diet, and this reduction power increased up to a 69.2% with the inclusion of concentrate in the 20:80 diet. The PE application significantly increased gas and carbon dioxide production and the concentration of ammonia-nitrogen, but decreased the pH (*p* < 0.001). In contrast, it did not interfere with organic matter and fiber digestibility. Supplementation with PE was effective in altering rumen fermentation toward less acetate and more propionate and butyrate (*p* < 0.001). Additionally, it improved the production of total volatile fatty acids in all feeding models (*p* < 0.001). In conclusion, the PE combination showed effective methane reduction by improving rumen fermentation characteristics without exhibiting adverse effects on fiber digestibility. Thus, PE could be used with all kinds of feeding models to effectively mitigate methane emissions from ruminants.

## 1. Introduction

Due to the continuous expansion of the world population, human demand for meat and milk is expected to increase by 73% and 58%, respectively, by 2050 compared with 2010 levels [1]. Therefore, to meet future needs, animal production must be increased. Although the livestock sector, especially ruminants, plays an essential role in food security, it is considered a significant source of greenhouse gases (GHGs), such as methane (CH_4_) and carbon dioxide (CO_2_), representing approximately 14–18% of global anthropogenic GHG emissions depending on the accounting approaches by various sources (IPCC, FAO, or others) [2]. These GHGs are directly related to global warming and climate change, which threaten the well-being of current and future generations [3]. Ruminants emit CH_4_ as a byproduct of their normal digestive process due to fermentation of feed. The CH_4_ released from enteric fermentation through eructation represents a loss of up to 15% of their gross energy intake, thus being one of the most important inefficiencies in ruminant production systems in addition to its environmental impact, since CH_4_ is 28 times more powerful than CO_2_ at trapping the sun’s heat [2,4].

Currently, studies are being performed of abatement strategies to reduce CH_4_ emissions and to improve the performance of ruminants. Through these studies, it has been proven that manipulation of the rumen microbiome with dietary supplements is one of the best mitigation strategies and that it has a two-sided benefit for both the environment and efficient livestock production [5,6]. Several research groups worldwide are investigating different kinds of feed additives/supplements with antimicrobial activity; however, the results reported in the literature are variable and show inconsistent efficacy [7,8]. One of the major reasons for the inconsistent effectiveness is related to the type of diet: forage- or concentrate-based diets [9]. It is often assumed that diet composition has a role in CH_4_ formation [10,11]. Therefore, there is an urgent need for a feed supplement that could achieve effective reduction of emissions with different kinds of ruminant diets without impairing rumen fermentation; meanwhile, supplements should be natural, as there is global interest in using plants and their secondary metabolites as alternatives to chemical compounds/antimicrobials in animal feed, and natural supplements are acceptable to consumers [12]. Nowadays, the potential of using phytochemicals gives the possibility of decreasing the negative impact of animal production on environment, but still, more effective supplements are required [6]. 

Plant-bioactives extract (PE), a mixture of organosulfur compounds extracted from garlic (*Allium sativum*) and flavonoids extracted from bitter orange (*Citrus aurantium*), showed the ability to reduce CH_4_ production without impairing rumen fermentation characteristics in two In Vitro studies [13,14]. We hypothesized that PE supplementation might have the same mode of action and the potential to reduce CH_4_ regardless of the grass:concentrate ratio. However, there are still limitations to proving the efficacy of this new combination with different feeding models (grass:concentrate ratios). Therefore, this study was conducted to investigate the potential of PE to be used as an antimethanogenic feed supplement with different feeding styles of ruminants, considering its impact on rumen fermentation and nutrient digestibility.

## 2. Materials and Methods 

### 2.1. Donor Animals and Rumen Fluid Collection 

The animals used in this experiment were kept and cared for by the Field Science Center, Obihiro University of Agriculture and Veterinary Medicine, Japan. The animal management and sampling procedures were approved by the Obihiro University of Agriculture and Veterinary Medicine, Animal Care and Use Committee (Approval number, 20-119). 

At 3 h after the morning feeding, approximately 3 L of rumen fluid was collected from two ruminally fistulated, nonlactating Holstein cows (880 kg average body weight). The cows were maintained on a daily diet of Orchard grass (*Dactylis glomerata*) hay (organic matter (OM), 980 g/kg; crude protein (CP), 132 g/kg; neutral detergent fiber (NDF), 701 g/kg; acid detergent fiber (ADF), 354 g/kg; acid detergent lignin (ADL), 40 g/kg; on dry matter (DM) basis) with free access to clean drinking water and mineral blocks (KOEN^®^ E250 TZ, Nippon Zenyaku Kogyo Co., Fukushima, Japan). The rumen fluid from each cow was collected from four different locations in the rumen. The collected rumen fluid was strained through four layers of surgical gauze into a thermos flask that was prewarmed to 39 °C and then immediately transferred to the laboratory within 15 min. 

### 2.2. Experimental Treatments and In Vitro Incubation Technique

Prior to the In Vitro incubation, ten experimental groups, with six replicates each, were prepared with approximately 500 mg (fresh matter) of ground substrate composed of Kleingrass (*Panicum coloratum*) hay and commercial concentrate mixture at different ratios with and without PE inclusion. The PE mixture was composed of 90% garlic granules (Allicin) and 10% citrus extract powder (Naringin, Naringenin, Neohesperidin, Rhoifolin, and Neoeriocitrin). The garlic powder used for PE preparation was sourced from cultivated and carefully processed and dried non-GMO garlic of Chinese origin. The dried garlic granules were standardized to contain 1% (w/w) allicin potential (S-Prop-2-en-1-yl prop-2-ene-1-sulfinothioate). Allicin concentration was determined by high performance liquid chromatography (HPLC) as described in details by Eger et al. [13]. The citrus components for the PE mixture (Naringin, Naringenin, Neohesperidin, Rhoifolin, and Neoeriocitrin) were developed from commercially available citrus extracts (Khush Ingredients, Oxford, United Kingdom) mainly extracted from bitter oranges (*Citrus aurantium*). The total polyphenol content of the citrus extract was standardized to 45% (w/w) by the Folin—Ciocalteau method [15]. Flavonoid concentrations were analyzed by HPLC using standards from (Sigma-Aldrich Ltd., Dorset, UK). Further information on the PE preparation was described in detail by Eger et al. [13]. This PE mixture is known commercially as Mootral and was provided by a Swiss company (Mootral SA, Rolle, Switzerland).

The experimental diets were as follows: 1- 1000 g grass/kg ration + 0 g concentrate/kg ration (100:0), 2- 100:0 + 200 g PE/kg of substrate (20 PE), 3- 80:20, 4- 80:20 + 20 PE, 5- 60:40, 6- 60:40 + 20 PE, 7- 40:60, 8- 40:60 + 20 PE, 9- 20:80, and 10- 20:80 + 20 PE. Five hundred milligrams of each of the experimental substrates (grass and concentrate) was added to preweighed ANKOM filter bags (F57, ANKOM Technology, Macedon, NY, USA), which were heat-sealed and placed in 120 mL glass bottles, whereas the PE mixture was added directly to the bottles (out of filter bag) one day before incubation. The dosage of PE used in the current study was based on the effective dose that altered the bacterial and archaeal communities in our previous In Vitro study [14]. Therefore, that effective dosage had to be used in further In Vitro trials for better understanding the mode of action of this new mixture. The chemical composition of the substrates and the PE are described in Table 1.

The procedure of In Vitro batch culture was performed as described by Menke and Steingass [16]. In the laboratory, the collected rumen fluids from the two cows were mixed together in one beaker under a constant stream of CO_2_. Forty milliliters of fresh buffer solution at a pH of 6.8 prepared according to McDougall [17] with twenty mL of rumen fluid was added to each 120 mL bottle under continuous CO_2_ flushing to maintain anaerobic conditions. Thereafter, the fermentation bottles were flushed with CO_2_ before sealing with butyl rubber stoppers and aluminum caps (Maruemu Co., Ltd, Osaka, Japan). All bottles were incubated for 24 h at 39 °C. This batch culture procedure was repeated in three consecutive runs on three different days. In each run, two blanks without substrate (empty filter bag plus 60 mL of buffered rumen fluid) were included to be used for digestibility and gas production correction. In total, 180 bottles plus 6 blank bottles were examined in this study.

### 2.3. Sample Collection

After 24 h of incubation, the total gas production was measured, and gas samples were collected from the headspace of the glass bottles into vacutainer tubes (BD Vacutainer^®^, Becton Drive, Franklin Lakes, NJ, USA). The tubes were stored at room temperature until CH_4_ and CO_2_ determination. Thereafter, the bottles’ caps were removed, and the pH of each tube was recorded using a pH meter (LAQUA F-72, HORIBA Scientific, Kyoto, Japan). Then, aliquots of the culture fluid were transferred into 1.5 mL Eppendorf tubes and centrifuged at 16,000× *g* and 4 °C for 5 min. The supernatant was collected and transferred into a new Eppendorf tube^®^ (Eppendorf AG, Hamburg, Germany), which was stored at −20 °C until use for volatile fatty acid (VFA) and ammonia nitrogen (NH_3_-N) analysis. The bags were removed from the bottles, washed under running tap water until the draining fluid became clear, and then dried at 60 °C for 48 h to determine the In Vitro dry matter digestibility (IVDMD). After IVDMD determination, the bags were used for the determination of In Vitro organic matter digestibility (IVOMD), In Vitro neutral detergent fiber digestibility (IVNDFD), and In Vitro acid detergent fiber digestibility (IVADFD). The residues in the fermentation bottles were discarded.

### 2.4. Chemical Analysis 

The chemical composition of the grass, concentrate, PE, and remaining substrate in the bags was determined following the standard procedure of AOAC [18]. The DM content was measured by drying the samples in an air-forced oven at 135 °C for 2 h (930.15). OM and ash were measured by placing the samples into a muffle furnace at 500 °C for 3 h (942.05). Nitrogen (N) was measured according to the method of Kjeldahl (984.13) using an electrical heating digester (DK 20, VELP Scientifica, Usmate (MB), Monza, Italy) and an automatic distillation apparatus (UDK 129 VELP Scientifica, Usmate (MB), Monza, Italy), and then CP was estimated as N × 6.25. The ADF, NDF, and ADL were measured and expressed as inclusive residual ash using an ANKOM^200^ Fiber Analyzer (Ankom Technology Methods 5, 6, and 8, respectively; ANKOM Technology Corp., Macedon, NY, USA). The NDF was measured using sodium sulfite with heat-stable α-amylase.

### 2.5. Gas Composition Analysis

The concentrations of CH_4_ and CO_2_ in the gas samples were determined by injection of 1 mL using a Hamilton gastight syringe (Hamilton Company, Reno, NV, USA) into a gas chromatograph (GC-8A, Shimadzu Corp., Kyoto, Japan). The carrier gas was helium. The temperatures of the infuser port, column, and detector were 70 °C, 150 °C, and 150 °C, respectively. The identification of CH_4_ and CO_2_ was based on the retention time.

### 2.6. Volatile Fatty Acids and Ammonia-Nitrogen Analysis

The concentration of VFA was determined using high-performance liquid chromatography (Shimadzu Corp., Kyoto, Japan) after diluting the supernatant 3 times with distilled water. Briefly, the analytical specifications were as follows: column, Shim-pak SCR-102H (7 mm, i.d. 8.0 mm × 300 mm, Shimadzu Corp., Kyoto, Japan); eluent flow rate and mobile phase for organic acid analysis (Shimadzu Corp., Kyoto, Japan) at 0.8 mL/min; column temperature, 40 °C; reaction reagent and flow rate, pH buffer for organic acid analysis (Shimadzu Corp., Kyoto, Japan) at 0.8 mL/min; conductivity detector (CDD-10AVP, Shimadzu Corp., Kyoto, Japan). Quantification of the VFA concentration was performed using an external standard quantitation method [14].

The NH_3_-N concentration was measured by diluting samples 50 times with 0.1 M phosphate buffer (pH 5.5) and then they were analyzed following the procedure of the modified Fujii—Okuda method [19] using an NH_3_ kit (FUJIFILM Wako Pure Chemical Corp, Osaka, Japan). The plate was read by a microplate reader (SH-1000 Lab, Corona Electric Co., Ltd., Japan) at an optical density of 630 nm.

### 2.7. Statistical Analysis

All variables were analyzed using PROC MIXED by SAS version 9.4 (SAS Institute Inc., Cary, NC, USA). The model included the treatment (diet) effect, PE effect, and their interaction as fixed effects, whereas the experimental runs were considered random effects. Least square means and standard error (SEM) were calculated, and the differences of means were estimated by pairwise *t*-tests (PDIFF option of PROC MIXED). Significance was declared at *p* < 0.05, and a tendency toward significance was declared when the *p* value was between 0.05 and 0.10.

## 3. Results

### 3.1. Effect of Plant-Bioactives Extract Supplementation on In Vitro pH, Gas Production, and Gas Composition

Supplementation of PE to all feeding models reduced the pH (*p* < 0.001) when compared with its corresponding group without PE supplementation in the same feeding model (Table 2). Moreover, the inclusion of PE increased the absolute total gas production when correlated with DM and digestible DM in all feeding styles (*p* < 0.001, Table 2).

Adding PE to all feeding styles decreased the proportion of CH_4_ but increased the proportion of CO_2_ in the produced gas (*p* < 0.001, Table 3). Furthermore, the CH_4_/CO_2_ ratio in the produced gas (mL/mL) decreased in all feeding models due to PE’s effect (*p* < 0.001, Table 3). The PE inclusion was effective with all diets in reducing the production of CH_4_/DM (mL/g) (*p* < 0.001); moreover, it reduced the production of CH_4_/digestible DM (mL/g) by 44.2%, 48.2%, 59.7%, 63.7%, and 69.2% in 100:0, 80:20, 60:40, 40:60, and 20:80, respectively, (*p* < 0.001). In contrast, the production of CO_2_/DM and CO_2_/digestible DM (mL/g) increased (*p* < 0.001) due to the effect of PE in all feeding styles (Table 3).

### 3.2. Effect of Plant-Bioactives Extract Supplementation on In Vitro Nutrient Digestibility and Ammonia-Nitrogen Concentration

The PE supplementation did not affect the IVDMD in the different experimental diets except in the 80:20 and 60:40 diets, where adding PE to these styles reduced the IVDMD (*p* < 0.01, Table 4). However, IVOMD, IVNDFD, and IVADFD did not show any differences when PE was added, as compared with their corresponding groups without PE supplementation (*p* > 0.05, Table 4). The PE inclusion increased the NH_3_-N concentration (*p* < 0.01) in 100:0 and 60:40, and it tended to increase in 40:60 (*p* = 0.088), but there was a non-significant numerical increase in 80:20 (*p* = 0.106) and 20:80 (*p* = 0.32) (Table 4).

### 3.3. Effect of Plant-Bioactives Extract Supplementation on In Vitro Volatile Fatty Acids

The PE supplementation did not show any effect on the acetate concentration in all feeding models; however, the interaction between PE and treatment showed a difference for the PE supplemented groups to be increased in 100:0 (*p* < 0.001) and to be decreased in 20:80 (*p* < 0.05) compared with its corresponding treatment without PE inclusion (Table 5). In contrast, the acetate ratio decreased in all feeding models due to PE supplementation (*p* < 0.01), whereas the interaction between PE and treatment did not have a significant effect (*p* > 0.05, Table 5). The concentration and the ratio of propionate and butyrate increased (*p* < 0.001) by adding PE in all feeding models. Additionally, the concentration of total volatile fatty acids (TVFA) showed the same finding (Table 5). The acetate/propionate (A/P) ratio decreased (*p* < 0.001) with PE supplementation in all feeding styles (Table 5).

## 4. Discussion

The CH_4_ emissions from ruminants are not only a serious environmental issue but also a significant source of energy loss to the animals. Different kinds of antimethanogenic compounds have already been studied to investigate their potential to reduce CH_4_ production [20]; however, there are limitations to their use due to their negative impacts on rumen fermentation characteristics [8], and they exhibited inconsistent efficiency with different feeding styles [9,21,22,23]. Therefore, sustainable and immediate CH_4_ mitigation strategies for the livestock industry are in high demand. Combining different plant extracts to achieve effective and sustainable CH_4_ reduction is a relatively new and promising approach [23]. The PE, a novel plant-based combination of garlic and citrus extracts, showed promising results when used as a feed supplement for CH_4_ mitigation from ruminants [13,24]. Therefore, this study was performed to evaluate the efficacy of PE with different kinds of feeding styles in ruminants. Although the current study provides interesting information about the effect of PE on rumen fermentation, digestibility, and CH_4_ production, the microbial analysis, which is very important for proper interpretation of the current findings, has not been done. Microbial characteristics are very important for obtaining the whole picture of fermentation, which was underlined by Pers-Kamczyc et al. [25]; however, our study, which we consider to be a pilot study, was carried out following the positive result of the tested PE effect on In Vitro rumen fermentation. Forthcoming research considering microbial analysis is strongly needed for a better understanding of the potential of this new mixture. Importantly, the finding that some of the differences in the results might arise from the differences in the nutrient composition due to PE addition rather than to the plant-bioactives present in PE must be taken into account when interpreting the current study.

Similar to the findings of the current study, PE increased gas production when used as a feed supplement with rumen fluid collected from sheep, which may reflect a stimulating effect of PE on rumen microbes [14]. This finding has been reported previously from a 48-h In Vitro gas production study conducted by Hansen and Nielsen [26]. Furthermore, PE increased the concentration of ruminal NH_3_-N, which might be due to the role of PE in enhancing the proteolysis process. This nitrogen source can be captured and used by rumen microorganisms to build their own protein [27], which in turn would be used as a protein source for the animal [28]. A similar effect has been reported when this PE was used as a feed supplement with a 70:30 ratio of forage to concentrate diet in the rumen simulation technique (RUSITEC) [29]. The same finding has also been observed with garlic oil with a 50:50 ratio of forage to concentrate diet for 24 h incubation by Busquet et al. [30]. 

The PE supplementation did not interfere with fiber degradability in all feeding models, which was similar to the findings of García-González et al. [31], who reported that inclusion of garlic bulbs in the substrate in an In Vitro trial did not affect IVNDFD, and Zhong et al. [32], who declared that adding garlic powder to the basal diet did not change the NDF and ADF digestibility through an in vivo trial using lambs. Rumen microbiome analysis in upcoming studies would provide a better understanding of PE’s effect on nutrient digestibility and proteolytic bacteria.

The synergism between the organosulfur compounds and flavonoids in the PE mixture was effective in decreasing CH_4_ production in all feeding models. The reduction in CH_4_ may be due to the direct inhibitory effect of PE on methanogenic archaea. In PE supplemented treatment, Eger et al. [13] and Ahmed et al. [14] reported a lower abundance of the family *Methanobacteriaceae,* which is the major CH_4_ producer in the rumen. This was attributed to the toxicity of organosulfur compounds of garlic, such as diallyl sulfide and allicin, in inhibiting certain sulfhydryl-containing enzymes essential for the metabolic activities of methanogenic archaea [33]. Moreover, it has been reported that flavonoids have the ability to reduce CH_4_ production as they have antimicrobial activities through interfering with cellular integrity of some bacteria as well as protozoa [34]. It has been established that ruminal ciliated protozoa could enhance methanogenesis, as they are major H_2_ producers in the rumen and are in symbiotic relationships with methanogens [35]. Although the impact of PE on protozoa has not yet been investigated, allicin and flavonoids have shown toxic effects on protozoa [36,37]. Any effect of PE on protozoa has to be confirmed in additional studies.

It is well established that CH_4_ formation has been positively associated with more acetate production and negatively associated with increased propionate production [38]. The allicin and flavonoids in PE were able to shift rumen fermentation toward less acetate and more propionate and butyrate. This increase in propionate may be due to the role of PE in increasing the abundance of the *Prevotellaceae* and *Veillonellaceae* families, which was confirmed by Ahmed et al. [14]. *Prevotellaceae* is one of the dominant families in rumen fluid, and it is well known to produce propionate by utilizing hydrogen (H_2_) produced during the fermentation of carbohydrates [39]. This pathway is the main pathway for H_2_ consumption, and it represents a competitive and alternative pathway to methanogenesis [40,41]. Moreover, the family *Veillonellaceae* showed high relative abundance due to the effect of flavonoids extracted from citrus [36], and it was associated with propionate production [42]. Supplementation of steers with garlic powder reduced the A/P ratio [43]. Similarly, the current study showed the same finding. An increase in butyrate was also associated with a reduction in CH_4_ production when the basal diet of ewes was supplemented with garlic extract [44]. 

Reports about the effects of garlic and flavonoid components on TVFA are inconsistent. Some studies reported that they had no effect on TVFA [31,45,46,47], but others reported an adverse effect [30,36,48] using an In Vitro batch culture system. In contrast, in the current study, the PE formulation increased the production of TVFA, which suggests improved feed efficiency. This phenomenon has also been observed previously in studies using In Vitro batch culture [14] and the RUSITEC system [13]. That improvement in TVFA production might have occurred because the produced H_2_ from fermentation was utilized by microorganisms to produce more propionate and butyrate in PE supplemented groups, while in control groups, part of H_2_ was utilized by methanogens to produce CH_4_ (energy loss). Therefore, the PE supplemented groups were more effective in redirecting H_2_ toward production of beneficial byproducts (energy source). Another theory is that PE stimulates the metabolic activity of some rumen microbes to utilize PE particles as feed, thus producing more TVFA—a theory that could be proven by demonstrating an increase in the production of total gas and CO2. The latter theory could also be supported by a recent report that flavonoids could be used as a source of carbon for metabolism in the rumen [34]. This finding has to be confirmed and discovered in upcoming research. 

## 5. Conclusions

In the present In Vitro study, we investigated the efficiency of the PE mixture on CH_4_ production, rumen fermentation, feed efficiency, and digestibility in different feeding styles. According to the design of this In Vitro study, PE at a level of 20%/substrate had the potential to effectively reduce CH_4_ production with all feeding styles. The PE showed a high reducing power up to 69% when the amount of concentrate composed up to 800 g/kg of the ration. Moreover, 20% PE supplementation improved the production of TVFA and shifted the fermentation profile toward less acetate and more propionate and butyrate. Additionally, PE did not impair fiber digestibility. Therefore, PE could be used as a feed supplement with all feeding styles to efficiently reduce CH_4_ production by ruminants. The hypothesis and design of this trial should be taken into consideration when interpreting the experimental results. The PE dosage used in the current In Vitro trial would not be feasible for practical feeding. Further long-term in vivo trials with optimum dosage have to be done to confirm the current findings.

## Figures and Tables

**Table 1:** Chemical composition of ration and plant-bioactives extract (g/kg of dry matter) used in 24 h In Vitro incubation.

(g/kg Dry Matter)	Kleingrass Hay	Concentrate	Plant-Bioactives Extract
Dry matter (g/kg fresh matter)	844.9	843.0	871.5
Organic matter	904.7	934.2	955.5
Crude ash	95.3	65.8	44.5
Crude protein	134.8	223.1	210.7
Ether extract	38.4	38.0	17.1
Neutral detergent fiber	662.5	232.6	35.5
Acid detergent fiber	362.6	109.1	33.7
Acid detergent lignin	52.2	30.1	1.8

**Table 2:** Effect of plant-bioactives extract (PE) supplementation on gas production and pH in different feeding styles after 24 h In Vitro incubation (*n* = 18).

Parameter	Treatments ^1^													
	100:0		80:20		60:40		40:60		20:80		SEM	*p*-Value		
	0	20	0	20	0	20	0	20	0	20		Trt	PE	Trt × PE
Gas production (mL)	30.06	45.53 ***	40.18	51.19 ***	43.50	53.08 ***	44.17	57.14 ***	45.94	54.47 ***	0.74	<0.001	<0.001	0.07
Gas/DM ^2^ (mL/g)	71.17	107.67 ***	94.90	120.93 ***	102.89	125.44 ***	104.31	135.09 ***	108.68	128.87 ***	1.76	<0.001	<0.001	0.07
Gas/Digestible DM (mL/g)	191.13	281.87 ***	207.46	288.09 ***	209.23	247.66 ***	200.41	266.66 ***	197.98	241.21 ***	3.18	<0.001	<0.001	<0.001
pH	6.58	6.50 ***	6.55	6.47 ***	6.53	6.44 ***	6.51	6.41 ***	6.50	6.40 ***	0.01	<0.001	<0.001	0.36

^1^ grass:concentrate ratio; 0: 0 g PE/kg; 20: 200 g PE/kg of substrate. ^2^ DM: dry matter. Asterisks in 20 mean significant difference between 0 and 200 g PE/kg in the same feeding model, *** (*p* < 0.001). Trt: treatment; PE: plant-bioactives extract; Trt × PE: interaction between treatment and plant-bioactives extract. SEM: standard error of the mean.

**Table 3:** Effect of plant-bioactives extract (PE) supplementation on CH_4_ and CO_2_ production in different feeding styles after 24 h In Vitro incubation (*n* = 18).

Parameter	Treatments ^1^													
	100:0		80:20		60:40		40:60		20:80		SEM	*p*-Value		
	0	20	0	20	0	20	0	20	0	20		Trt	PE	Trt × PE
CH_4_ (%)	5.19	1.99 ***	5.59	2.07 ***	5.87	1.84 ***	5.83	1.59 ***	5.89	1.51 ***	0.16	0.006	<0.001	<0.001
CO_2_ (%)	94.81	98.01 ***	94.41	97.93 ***	94.13	98.16 ***	94.17	98.41 ***	94.11	98.49 ***	0.16	0.006	<0.001	0.21
CH_4_/CO_2_ ratio (mL/mL)	0.055	0.020 ***	0.059	0.021 ***	0.062	0.019 ***	0.062	0.016 ***	0.063	0.015 ***	0.002	0.004	<0.001	<0.001
CH_4_/DM ^2^ (mL/g)	3.68	2.09 ***	5.36	2.57 ***	6.11	2.30 ***	6.09	2.16 ***	6.48	1.91 ***	0.16	<0.001	<0.001	<0.001
CO_2_/DM (mL/g)	67.45	105.58 ***	89.50	118.36 ***	96.78	123.14 ***	98.22	132.93 ***	102.20	126.40 ***	1.78	<0.001	<0.001	0.12
CH_4_/digestible DM (mL/g)	9.88	5.51 ***	11.62	6.02 ***	12.35	4.98 ***	11.64	4.23 ***	11.66	3.59 ***	0.29	<0.001	<0.001	<0.001
CO_2_/digestible DM (mL/g)	181.26	276.36 ***	195.85	282.07 ***	196.89	269.68 ***	188.77	262.43 ***	186.33	237.60 ***	3.35	<0.001	<0.001	<0.001

^1^ grass:concentrate ratio; 0: 0 g PE/kg; 20: 200 g PE/kg of substrate. ^2^ DM: dry matter. Asterisks in 20 mean significant difference between 0 and 200 g PE/kg in the same feeding model, *** (*p* < 0.001). Trt: treatment; PE: plant-bioactives extract; Trt × PE: interaction between treatment and plant-bioactives extract. SEM: standard error of the mean.

**Table 4:** Effect of plant-bioactives extract (PE) supplementation on digestibility and ammonia-nitrogen in different feeding styles after 24 h In Vitro incubation (*n* = 18).

Parameter	Treatments ^1^													
	100:0		80:20		60:40		40:60		20:80		SEM	*p*-Value		
	0	20	0	20	0	20	0	20	0	20		Trt	PE	Trt × PE
IVDMD ^2^	0.38	0.38	0.45	0.42 **	0.49	0.46 **	0.52	0.51	0.55	0.53	0.56	<0.001	0.001	0.10
IVOMD ^3^	0.43	0.41	0.51	0.49	0.57	0.52	0.59	0.59	0.64	0.61	0.02	<0.001	0.03	0.82
IVNDFD ^4^	0.36	0.36	0.40	0.38	0.38	0.32	0.36	0.34	0.32	0.35	0.01	0.61	0.56	0.76
IVADFD ^5^	0.21	0.24	0.25	0.21	0.21	0.23	0.20	0.23	0.22	0.23	0.01	0.97	0.62	0.64
NH_3_-N ^6^ (mg/dL)	4.46	6.43 ***	6.30	7.09	6.14	7.51 **	6.01	6.84	7.35	7.83	0.18	<0.001	<0.001	0.17

^1^ grass:concentrate ratio; 0: 0 g PE/kg; 20: 200 g PE/kg of substrate. ^2^ IVDMD: In Vitro dry matter digestibility. ^3^ IVOMD: In Vitro organic matter digestibility. ^4^ IVNDFD: In Vitro neutral detergent fiber digestibility. ^5^ IVADFD: In Vitro acid detergent fiber digestibility. ^6^ NH_3_-N: ammonia-nitrogen. Asterisks in 20 mean significant difference between 0 and 200 g PE/kg in the same feeding model, ** (*p* < 0.01), *** (*p* < 0.001). Trt: treatment; PE: plant-bioactives extract; Trt × PE: interaction between treatment and plant-bioactives extract. SEM: standard error of the mean.

**Table 5:** Effect of plant-bioactives extract (PE) supplementation on volatile fatty acids in different feeding styles after 24 h In Vitro incubation (*n* = 18).

Parameter	Treatments ^1^													
	100:0		80:20		60:40		40:60		20:80		SEM	*p*-Value		
	0	20	0	20	0	20	0	20	0	20		Trt	PE	Trt × PE
Acetate (mmol/L)	62.54	66.02 **	64.81	66.12	64.81	65.66	65.61	65.91	66.80	64.15 *	0.54	0.45	0.21	0.01
Propionate (mmol/L)	15.24	20.19 ***	18.57	22.95 ***	19.38	24.34 ***	20.61	26.82 ***	22.21	26.78 ***	0.29	<0.001	<0.001	0.20
Butyrate (mmol/L)	7.14	10.86 ***	8.53	11.38 ***	9.01	12.36 ***	9.17	12.71 ***	9.84	12.89 ***	0.16	<0.001	<0.001	0.21
TVFA ^2^ (mmol/L)	84.91	97.07 ***	91.92	100.45 ***	93.20	102.36 ***	95.40	105.44 ***	98.85	103.81 *	0.78	<0.001	<0.001	0.07
Acetate (mol/100 mol)	73.64	67.93 ***	70.53	65.67 ***	69.47	64.00 ***	68.72	62.39 ***	67.54	61.59 ***	0.30	<0.001	<0.001	0.07
Propionate (mol/100 mol)	17.95	20.80 ***	20.20	22.92 ***	20.86	23.86 ***	21.66	25.49 ***	22.46	25.92 ***	0.20	<0.001	<0.001	0.04
Butyrate (mol/100 mol)	8.41	11.27 ***	9.27	11.41 ***	9.67	12.14 ***	9.62	12.12 ***	10.00	12.50 ***	0.13	<0001	<0.001	0.50
A/P ratio ^3^	4.11	3.29 ***	3.49	2.88 ***	3.34	2.69 ***	3.19	2.46 ***	3.02	2.40 ***	0.04	<0.001	<0.001	0.03

^1^ grass:concentrate ratio; 0: 0 g PE/kg; 20: 200 g PE/kg of substrate. ^2^ TVFA: total volatile fatty acids. ^3^ A/P: acetate/propionate. Asterisks in 20 mean significant difference between 0 and 200 g PE/kg in the same feeding model, * (*p* < 0.05), ** (*p* < 0.01), *** (*p* < 0.001). Trt: treatment; PE: plant-bioactives extract; Trt × PE: interaction between treatment and plant-bioactives extract. SEM: standard error of the mean.

## Data Availability

The data supporting reported results in this study are available on request from the corresponding author.

## References

[1] Grossi G., Goglio P., Vitali A., Williams A.G. (2019). Livestock and climate change: Impact of livestock on climate and mitigation strategies. Anim. Front..

[2] Gerber P.J., Steinfeld H., Henderson B., Mottet A., Opio C., Dijkman J., Falcucci A., Tempio G. (2013). Tackling Climate Change through Livestock: A Global Assessment of Emissions and Mitigation Opportunities.

[3] Scholtz M.M., Neser F.W., Makgahlela M.L. (2020). A balanced perspective on the importance of extensive ruminant production for human nutrition and livelihoods and its contribution to greenhouse gas emissions. S. Afr. J. Sci..

[4] Johnson K.A., Johnson D.E. (1995). Methane emissions from cattle. J. Anim. Sci..

[5] Bryszak M., Szumacher-Strabel M., Huang H., Pawlak P., Lechniak D., Kołodziejski P., Yanza Y.R., Patra A.K., Váradyová Z., Cieslak A. (2020). *Lupinus angustifolius* seed meal supplemented to dairy cow diet improves fatty acid composition in milk and mitigates methane production. Anim. Feed Sci. Technol..

[6] Honan M., Feng X., Tricarico J.M., Kebreab E. (2021). Feed additives as a strategic approach to reduce enteric methane production in cattle: Modes of action, effectiveness and safety. Anim. Prod. Sci..

[7] Fagundes G.M., Benetel G., Carriero M.M., Sousa R.L.M., Muir J.P., Macedo R.O., Bueno I.C.S. (2021). Tannin-rich forage as a methane mitigation strategy for cattle and the implications for rumen microbiota. Anim. Prod. Sci..

[8] Jafari S., Ebrahimi M., Goh Y.M., Rajion M.A., Jahromi M.F., Al-Jumaili W.S. (2019). Manipulation of rumen fermentation and methane gas production by plant secondary metabolites (saponin, tannin and essential oil)—A review of ten-year studies. Ann. Anim. Sci..

[9] Vázquez-Carrillo M.F., Montelongo-Pérez H.D., González-Ronquillo M., Castillo-Gallegos E., Castelán-Ortega O.A. (2020). Effects of three herbs on methane emissions from beef cattle. Animals.

[10] Van Lingen H.J., Niu M., Kebreab E., Valadares Filho S.C., Rooke J.A., Duthie C.-A., Schwarm A., Kreuzer M., Hynd P.I., Caetano M. (2019). Prediction of enteric methane production, yield and intensity of beef cattle using an intercontinental database. Agric. Ecosyst. Environ..

[11] Hernandez-De Lira I.O., Huber D.H., Espinosa-Solares T., Balagurusamy N. (2015). Methane emission and bioenergy potential from livestock manures in Mexico. J. Renew. Sustain. Energy.

[12] Ku-Vera J.C., Jiménez-Ocampo R., Valencia-Salazar S.S., Montoya-Flores M.D., Molina-Botero I.C., Arango J., Gómez-Bravo C.A., Aguilar-Pérez C.F., Solorio-Sánchez F.J. (2020). Role of secondary plant metabolites on enteric methane mitigation in ruminants. Front. Vet. Sci..

[13] Eger M., Graz M., Riede S., Breves G. (2018). Application of Mootral^TM^ reduces methane production by altering the archaea community in the rumen simulation technique. Front. Microbiol..

[14] Ahmed E., Yano R., Fujimori M., Kand D., Hanada M., Nishida T., Fukuma N. (2021). Impacts of Mootral on methane production, rumen fermentation, and microbial community in an In Vitro study. Front. Vet. Sci..

[15] Kaur C., Kapoor H.C. (2002). Anti-oxidant activity and total phenolic content of some Asian vegetables. Int. J. Food Sci. Technol..

[16] Menke K.H., Steingass H. (1988). Estimation of the energetic feed value obtained from chemical analyses and gas production using rumen fluid. Anim. Res. Dev..

[17] Mcdougall E.I. (1948). The composition and output of sheep’s saliva. Biochem. J..

[18] AOAC (1995). Official Methods of Analysis.

[19] Kawasaki K., Min X., Li X., Hasegawa E., Sakaguchi E. (2015). Transfer of blood urea nitrogen to cecal microbial nitrogen is increased by fructo-oligosaccharide feeding in guinea pigs. Anim. Sci. J..

[20] Patra A., Park T., Kim M., Yu Z. (2017). Rumen methanogens and mitigation of methane emission by anti-methanogenic compounds and substances. J. Anim. Sci. Biotechnol..

[21] Lee S.J., Lee Y.J., Eom J.S., Kim H.S., Choi Y.Y., Jo S.U., Kang S.N., Park H.Y., Kim D.H., Lee S.S. (2020). Effects of the appropriate addition of antioxidants from *Pinus densiflora* and *Mentha canadensis* extracts on methane emission and rumen fermentation. Animals.

[22] Suybeng B., Charmley E., Gardiner C.P., Malau-Aduli B.S., Malau-Aduli A.E.O. (2020). Supplementing Northern Australian beef cattle with desmanthus tropical legume reduces in-vivo methane emissions. Animals.

[23] Jayanegara A., Yogianto Y., Wina E., Sudarman A., Kondo M., Obitsu T., Kreuzer M. (2020). Combination effects of plant extracts rich in tannins and saponins as feed additives for mitigating In Vitro ruminal methane and ammonia formation. Animals.

[24] Roque B.M., van Lingen H.J., Vrancken H., Kebreab E. (2019). Effect of Mootral—A garlic- and citrus-extract-based feed additive—On enteric methane emissions in feedlot cattle. Transl. Anim. Sci..

[25] Pers-Kamczyc E., Zmora P., Cieślak A., Szumacher-Strabel M. (2011). Development of nucleic acid based techniques and possibilities of their application to rumen microbial ecology research. J. Anim. Feed Sci..

[26] Hansen H., Nielsen M. (2018). Impact of Mootral on Rumen Digestion. University of Copenhagen. https://ivh.ku.dk/nyheder/2018/impact-of-mootral-on-rumen-digestion/.

[27] Marcos C.N., Carro M.D., Fernández-Yepes J.E., Arbesu L., Molina-Alcaide E. (2020). Utilization of Avocado and Mango fruit wastes in multi-nutrient blocks for goats feeding: In Vitro evaluation. Animals.

[28] Wang R., Wang M., Ungerfeld E.M., Zhang X.M., Long D.L., Mao H.X., Deng J.P., Bannink A., Tan Z.L. (2018). Nitrate improves ammonia incorporation into rumen microbial protein in lactating dairy cows fed a low-protein diet. J. Dairy Sci..

[29] Brede J., Eger M., Breves G. Dose-dependent effects of a garlic-citrus powder on methane production and fermentation parameters of rumen microbial metabolism. Proceedings of the XIIIth International Symposium on Ruminant Physiology (ISRP).

[30] Busquet M., Calsamiglia S., Ferret A., Carro M.D., Kamel C. (2005). Effect of garlic oil and four of its compounds on rumen microbial fermentation. J. Dairy Sci..

[31] García-González R., López S., Fernández M., Bodas R., González J.S. (2008). Screening the activity of plants and spices for decreasing ruminal methane production In Vitro. Anim. Feed Sci. Technol..

[32] Zhong R., Xiang H., Cheng L., Zhao C., Wang F., Zhao X., Fang Y. (2019). Effects of feeding garlic powder on growth performance, rumen fermentation, and the health status of lambs infected by gastrointestinal nematodes. Animals.

[33] Patra A.K., Yu Z. (2015). Effects of adaptation of In Vitro rumen culture to garlic oil, nitrate, and saponin and their combinations on methanogenesis, fermentation, and abundances and diversity of microbial populations. Front. Microbiol..

[34] Hassan F.-U., Arshad M.A., Li M., Rehman M.S.-U., Loor J.J., Huang J. (2020). Potential of Mulberry leaf biomass and its flavonoids to improve production and health in ruminants: Mechanistic insights and prospects. Animals.

[35] Levy B., Jami E. (2018). Exploring the prokaryotic community associated with the rumen ciliate protozoa population. Front. Microbiol..

[36] Oskoueian E., Abdullah N., Oskoueian A. (2013). Effects of flavonoids on rumen fermentation activity, methane production, and microbial population. BioMed Res. Int..

[37] Miron T., Rabinkov A., Mirelman D., Wilchek M., Weiner L. (2000). The mode of action of allicin: Its ready permeability through phospholipid membranes may contribute to its biological activity. Biochim. Biophys. Acta Biomembr..

[38] Vargas J.E., Andrés S., López-Ferreras L., Snelling T.J., Yáñez-Ruíz D.R., García-Estrada C., López S. (2020). Dietary supplemental plant oils reduce methanogenesis from anaerobic microbial fermentation in the rumen. Sci. Rep..

[39] Denman S.E., Martinez Fernandez G., Shinkai T., Mitsumori M., McSweeney C.S. (2015). Metagenomic analysis of the rumen microbial community following inhibition of methane formation by a halogenated methane analog. Front. Microbiol..

[40] Ungerfeld E.M. (2015). Shifts in metabolic hydrogen sinks in the methanogenesis-inhibited ruminal fermentation: A meta-analysis. Front. Microbiol..

[41] Wang K., Nan X., Chu K., Tong J., Yang L., Zheng S., Zhao G., Jiang L., Xiong B. (2018). Shifts of hydrogen metabolism from methanogenesis to propionate production in response to replacement of forage fiber with non-forage fiber sources in diets In Vitro. Front. Microbiol..

[42] Chen L., Shen Y., Wang C., Ding L., Zhao F., Wang M., Fu J., Wang H. (2019). *Megasphaera elsdenii* lactate degradation pattern shifts in rumen acidosis models. Front. Microbiol..

[43] Wanapat M., Khejornsart P., Pakdee P., Wanapat S. (2008). Effect of supplementation of garlic powder on rumen ecology and digestibility of nutrients in ruminants. J. Sci. Food Agric..

[44] Ma T., Chen D., Tu Y., Zhang N., Si B., Deng K., Diao Q. (2016). Effect of supplementation of allicin on methanogenesis and ruminal microbial flora in Dorper crossbred ewes. J. Anim. Sci. Biotechnol..

[45] Busquet M., Calsamiglia S., Ferret A., Cardozo P.W., Kamel C. (2005). Effects of cinnamaldehyde and garlic oil on rumen microbial fermentation in a dual flow continuous culture. J. Dairy Sci..

[46] Seradj A., Abecia L., Crespo J., Villalba D., Fondevila M., Balcells J. (2014). The effect of Bioflavex^®^ and its pure flavonoid components on In Vitro fermentation parameters and methane production in rumen fluid from steers given high concentrate diets. Anim. Feed Sci. Technol..

[47] Klevenhusen F., Zeitz J.O., Duval S., Kreuzer M., Soliva C.R. (2011). Garlic oil and its principal component diallyl disulfide fail to mitigate methane, but improve digestibility in sheep. Anim. Feed Sci. Technol..

[48] Dey A., Paul S.S., Lailer P.C., Dahiya S.S. (2021). Reducing enteric methane production from buffalo (*Bubalus bubalis*) by garlic oil supplementation in In Vitro rumen fermentation system. SN Appl. Sci..

